# Intraepithelial lymphocytes are indicators of better prognosis in surgically resected endometrioid-type endometrial carcinomas at early and advanced stages

**DOI:** 10.1186/s12885-022-09363-0

**Published:** 2022-04-02

**Authors:** Takako Kono-Sato, Kosuke Miyai, Yoji Yamagishi, Morikazu Miyamoto, Masashi Takano, Susumu Matsukuma, Kimiya Sato, Hitoshi Tsuda

**Affiliations:** 1 Department of Basic Pathology, National Defense Medical College, Tokorozawa 359-8513, Saitama, Japan; 2 Department of Pathology and Laboratory Medicine, National Defense Medical College, Tokorozawa, Japan; 3 Department of Obstetrics and Gynecology, National Defense Medical College Hospital, Tokorozawa, Japan

**Keywords:** Endometrial cancer, Endometrioid carcinoma, Tumor-infiltrating lymphocyte, Tumor-associated macrophage, DNA mismatch repair deficiency

## Abstract

**Background:**

Tumor-infiltrating lymphocytes (TILs) and tumor-associated macrophages (TAMs) may be useful prognostic indicators in endometrial cancer. However, standardized assessment methods and the prognostic roles of these cells in different stage groups are unclear.

**Methods:**

Formalin-fixed paraffin-embedded tissue samples of 107 endometrioid-type endometrial carcinomas (EECs) comprising 60 stage IB and 47 stage IIIC or IVB cases were evaluated. CD3^+^ TILs, CD8^+^ TILs, CD68^+^ TAMs, and CD163^+^ TAMs were detected by immunohistochemistry, and their densities were evaluated by semiquantitative and quantitative methods. TILs within tumor epithelial cell nests (E-TILs) and those within the stroma at the invasive front (S-TILs) were evaluated separately for CD3^+^ and CD8^+^ cells. The “TIL score” was defined as the sum of semiquantitative scores of CD3^+^ E-TILs, CD3^+^ S-TILs, CD8^+^ E-TILs, and CD8^+^ S-TILs. For TAMs, the area of CD68^+^ and CD163^+^ cells in the invasive margin were semiquantitatively and quantitatively evaluated. Clinicopathological and prognostic implications of TILs and TAMs in stage IB and IIIC/IVB EECs were examined by Cox univariate and multivariate analyses.

**Results:**

By Cox univariate analyses, semiquantitatively low CD3^+^ E-TILs, low CD8^+^ E-TILs, and low “TIL score” were significantly correlated with worse prognosis in stage IB patients (*P* = 0.011, 0.040, and 0.039, respectively). Likewise, low CD3^+^ E-TILs and low CD8^+^ E-TILs, by both semiquantitative (*P* = 0.011 and 0.0051) and quantitative evaluations (*P* < 0.0001, and *P* = 0.0015) and low “TIL score” (*P* = 0.020) were significantly correlated with worse prognosis in stage IIIC/IVB patients. By Cox multivariate analyses, semiquantitatively low CD3^+^ E-TILs and low CD8^+^ E-TILs, low “TIL score”, and quantitatively low CD3^+^ E-TILs and low CD8^+^ E-TILs were independent worse prognostic factors in stage IIIC/IVB (*P* = 0.0011, 0.0053, 0.012*,* < 0.0001, and < 0.0001, respectively). CD68^+^ or CD163^+^ TAMs were not correlated with prognosis in any patients.

**Conclusions:**

Both semiquantitatively and quantitatively low E-TILs, are correlated with worse prognosis in both early and advanced stage patients with EECs. In particular, CD3^+^ E-TILs and CD8^+^ E-TILs are potentially useful prognostic markers in patients with EEC regardless of the stage.

**Supplementary Information:**

The online version contains supplementary material available at 10.1186/s12885-022-09363-0.

## Background

Endometrial cancer (EC) is one of the most common gynecological malignancies; in 2018, there were 382,069 new cases and 89,929 deaths worldwide [1]. The incidence of EC in Asian women is increasing, particularly in postmenopausal women [2], with 11,120 novel cases in 2017 and 2601 deaths reported in Japan in 2018 [3, 4]. In EC, endometrioid-type endometrial carcinoma (EEC) is the most prevalent histological type with excellent clinical outcomes in the earlier stage diseases, but the outcomes get worse in more advanced stages. For example, 5-year overall survival (OS) rates were reported to be 95.7 and 89.3% in International Federation of Gynecology and Obstetrics (FIGO) stage I and II patients but 81.9 and 37.2% in FIGO stage III and IV patients, respectively, in Japan [3].

Recently, tumor immune cells, such as tumor-infiltrating lymphocytes (TILs) and tumor-associate macrophages (TAMs), have been shown to be of prognostic significance in EC [5]: a larger number of TILs, detected immunohistochemically as CD3^+^ and CD8^+^ T cells, were associated with better prognosis [5–8]. Most previous studies [5] have investigated TILs within tumor epithelial cell nests (epithelial TILs, E-TILs) and those within the stroma at the invasive front (stromal TILs, S-TILs) [9]. In EC, CD8^+^ E-TILs showed prognostic implications [5–10], but the prognostic roles of CD3^+^ E-TILs, CD3^+^ S-TILs and CD8^+^ S-TILs remain controversial [5, 7–9].

In colorectal cancer, a powerful prognostication tool known as the “Immunoscore” was developed [11, 12]. The Immunoscore is a digital image analysis system for evaluating the immunohistochemical density of CD3^+^ and CD8^+^ lymphocytes in the center and invasive margin of the tumor. A similar approach for EC may be useful, as EC and colorectal cancer have common histology and major prognostic features, including depth of invasion, lymph node metastasis, and lymphovascular invasion (LVI).

TAMs have also been examined by many study groups. There are two types of effector macrophages, type I (M1) and type II (M2**)**. M1 macrophages play tumor-suppressive roles including killing of pathogens and tumor cells, whereas M2 macrophages have tumor-promoting roles by inhibiting inflammation, suppressing T-cell function, and promoting angiogenesis [13, 14]. TAMs mainly consist of the M2 type, and abundant M2 infiltration in cancer tissue was reported to indicate poor prognosis, although there are equivocal data among the reports and tumor types [5, 15–17].

In previous studies of TILs and TAMs in EC, most patients were in early stages, i.e., I and II, and all histological types were included. Few separate analyses of TILs in earlier and advanced stages in EC have been performed. The FIGO stage classification is not perfect for predicting clinical outcomes: in patients with stage I EC, the 5-year survival rate is > 90%, but deeper myometrial invasion, grade 3, and lymphovascular invasion (LVI) are shown to be factors of high risk [18]. Identifying additional powerful prognostic indicators would be important for more appropriate management of stage I patients. Stratification into subgroups with different prognosis of patients with advanced stage ECs could also be of clinical value.

In the present study, we immunohistochemically examined TILs and TAMs in surgically resected ECs to reveal their clinical roles in early and advanced stage groups. Both semiquantitative and quantitative approaches were used to evaluate TILs and TAMs to reveal their optimal measurements. Because EC contains various histological types with different biological properties, we focused on EEC.

## Methods

This was a retrospective study performed in a single institute.

### Ethics approval and consent to participate

This study was performed in accordance with the Declaration of Helsinki and was approved by the institutional review board of National Defense Medical College (registration number: 2516). Written informed consent was obtained from all patients.

### Patients

Of the 556 patients with EC treated by the primary surgery including hysterectomy at the National Defense Medical College Hospital between 1990 and 2014, 464 patients were diagnosed with EEC. Of these, 73, 45, and 17 patients were in stage IB, IIIC, and IVB, respectively, according to FIGO 2008. Archival paraffin-embedded tumor blocks or clinical data were not available for 24 patients (12 IB, 7 IIIC, and 5 IVB), and other four patients were excluded because of non-EEC histology (1 IB carcinosarcoma and 1 IIIC and 2 IVB serous carcinomas) following histological review. Finally, a total of 107 cases were enrolled in this study: 60 IB patients as the early stage group and 37 IIIC and 10 IVB patients as the advanced stage group. We did not include stage IA and II patients in the study because of almost no events and a small number of cases, respectively.

All patients were Asian. No patients underwent radiation or chemotherapy before surgical therapy. Ninety patients received adjuvant chemotherapy and**/**or radiation, whereas the other 17 patients, comprising 14 stage IB, 2 stage IIIC, and 1 stage IVB patients, were not treated with additional adjuvant therapies for various reasons.

### Immunohistochemistry

Among the 107 EECs, one representative paraffin-embedded primary tumor block was selected. The blocks were cut into 4-μm-thick sections and subjected to immunohistochemistry. The antibodies used were rabbit polyclonal anti-CD3 (DAKO/Agilent, Santa Clara, CA, USA), mouse monoclonal anti-CD8 (C8/144B, Nichirei, Tokyo, Japan), mouse monoclonal anti-CD68 (PG-M1, DAKO/Agilent), mouse monoclonal anti-CD163 (10D6, Leica, Wetzlar, Germany), mouse monoclonal anti-MLH-1 (ES05, DAKO/Agilent), mouse monoclonal anti-MSH-2 (FE11, DAKO/Agilent), rabbit monoclonal anti-MSH-6 (EPR3945, GeneTex, Los Angeles, CA, USA), and rabbit monoclonal anti-PMS-2 (EP51, DAKO/Agilent).

After appropriate antigen retrieval, endogenous peroxidase was blocked with 0.3% hydrogen peroxidase in methanol, and nonspecific staining was blocked with skim milk. The slides were incubated with primary antibodies at 4 °C overnight and, then with biotinylated secondary antibody (DAKO REAL Envision) at room temperature for 30 min, followed by staining with 3,3-diaminobenzidine (Muto Pure Chemicals, Tokyo, Japan) and counterstained with Mayer’s hematoxylin.

DNA mismatch repair (MMR) was judged as deficient if one or more of the MLH-1, MSH-2, MSH-6, and PMS-2 protein expressions were completely negative. Otherwise, MMR was designated as proficient.

Two independent observers (T.K-S. and H.T.) counted the number of immunostained TILs and TAMs in tumor tissue without prior knowledge of clinical information. Any discrepancies between the two observers were resolved by discussion.

#### Evaluations of TILs and TAMs

E-TILs and S-TILs identified by CD3 and CD8 immunoreactivities were evaluated using semiquantitative and quantitative methods, both of which employed manual counting. In semiquantitative method, the degrees of S-TILs and TAMs infiltration within the invasive margin were evaluated under the light microscope and stratified into three categories. The degree of E-TILs infiltration within the tumor cell nests was also evaluated under the microscope into these three categories, but semiquantitative measurement of E-TILs per HPF was also employed. In these semiquantitative evaluations, essentially discontinuous categories were analyzed.

In quantitative evaluation, the number of immunopositive cells per field on digital slides was manually counted for TILs, and the ratio of immunopositive area per stromal area on digital slides was automatically counted for TAMs. In these quantitative evaluations, continuous values were analyzed.

##### Semiquantitative evaluations of E-TILs, S-TILs, and “TIL score”

**Fig. 1:**
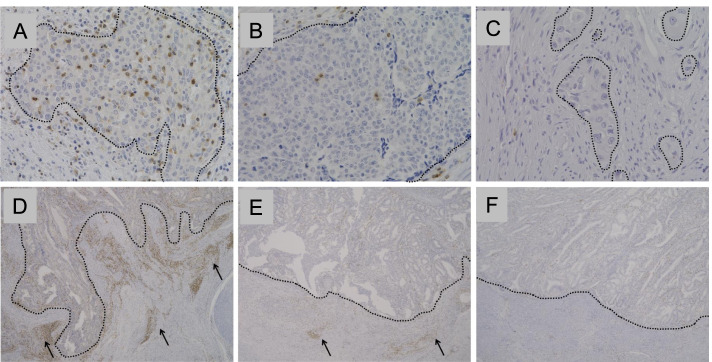
Semiquantitative evaluation of epithelial tumor-infiltrating lymphocytes (E-TILs) and stromal tumor-infiltrating lymphocytes (S-TILs). **A**–**C** Representative cases of CD3^+^ E-TILs, **A** high: > 10 immunopositive lymphocyte per high-power field (HPF) (×400) within the tumor cell nests, **B** intermediate: between high and low, **C** low: < 3 immunopositive lymphocytes per HPF, ×400. **D**–**F** Representative cases of CD3^+^ S-TILs, **D** high: zonal dense infiltration in over half length of the invasive front stroma area (arrows), **E** intermediate: focal nested infiltration (arrows), **F** low: entirely sparse infiltration (< 10% length), × 40

Under low to intermediate magnification using a BX-70 microscope (Olympus, Tokyo, Japan), the density of E-TILs and S-TILs was classified as low (score 0), intermediate (score 1), or high (score 2). To evaluate the E-TILs densities, we also referred to the average number of immunopositive lymphocytes on the tumor cell nests per high-power field (HPF) (× 400): E-TILs were considered as high, intermediate, and low when > 10, 3–10, and < 3 immunopositive lymphocytes were observed, respectively (Fig. 1A–C). The thresholds (3 and 10/HPF) were determined on our assumption by referring in part to the study of Kondratiev et al. [6]. High, intermediate, and low S-TILs densities were determined when infiltration of immunopositive lymphocytes was observed in over half length (band-like), focal to less than half the length (10 to < 50%), and sparse or absent (< 10%), respectively, of the invasive front stromal area (Fig. 1D–F). These thresholds were determined by referring in part to the study of Yamashita et al. [19].

As observed for the “Immunoscore” in colorectal cancer [11, 12], a combination of evaluations of CD3^+^ TILs and CD8^+^ TILs may be a more powerful prognostic indicator than solitary evaluation. Therefore, a simplified “TIL score” was assigned to all cases. The TIL score comprised the sum of semiquantitative points of four TILs, i.e., CD3^+^ E-TILs, CD3^+^ S-TILs, CD8^+^ E-TILs, and CD8^+^ S-TILs. Low, intermediate, and high TILs were assigned as point 0, 1, and 2, respectively, and the scores as the sum of points ranged from 0 to 8.

##### Quantitative evaluation of E-TILs and S-TILs

For E-TILs, three representative HPFs (× 400) were selected, photomicrographs were acquired as digital images, and from the images, the average numbers of CD3^+^ TILs and CD8^+^ TILs per HPF (0.238 mm^2^) on the tumor cell nests were counted manually. For S-TILs, five representative HPFs in the invasive front were selected because S-TILs tended to be distributed more heterogeneously than E-TILs, and the average number of CD3^+^ TILs and CD8^+^ TILs in the stroma per HPF was counted manually.

#### TAMs

**Fig. 2:**
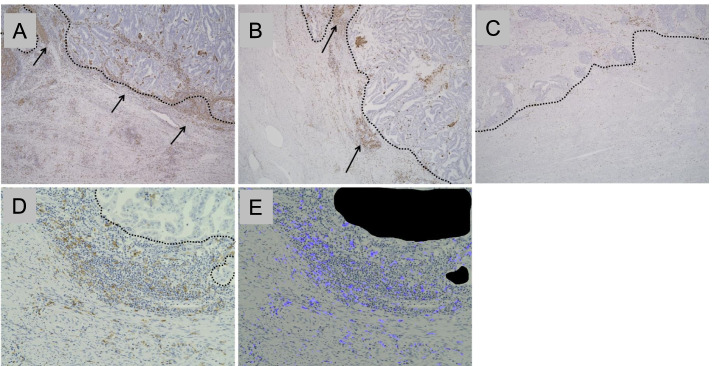
Semiquantitative and quantitative evaluation of tumor associated macrophages (TAMs). **A**–**C** Representative cases of CD163^+^ TAMs, **A** high: zonal dense infiltration (arrows), **B** intermediate: focal nested infiltration (arrows), **C** low: entirely sparse infiltration, × 40. **D**, **E** Example case of quantitative evaluation of CD163^+^ TAMs using a BZ-X-700 microscope (Keyence), **D** original photo of immunohistochemical staining for CD163, **E** $\mathrm{CD}163\ \mathrm{positive}\ \mathrm{TAMs}\ \mathrm{area}\ \mathrm{ratio}=\frac{\mathrm{CD}163\ \mathrm{positive}\ \mathrm{TAMs}\ \mathrm{area}}{\mathrm{background}\ \mathrm{stroma}\ \mathrm{area}}=\frac{\mathrm{blue}\ \mathrm{area}}{\mathrm{background}-\mathrm{black}\ \left(\mathrm{tumor}\right)\ \mathrm{area}}$

We semiquantitatively evaluated the density of CD68^+^ and CD163^+^ cells in the invasive fronts of the tumor with low- to intermediate-power magnification and classified the density as high, intermediate, or low as described for S-TILs (Fig. 2A–C). The thresholds were determined by referring to semiquantitative S-TILs evaluation method above. For quantitative evaluation, three representative intermediate-power fields (× 200) were selected, and the area ratios (%) of CD68^+^ and CD163^+^ cells to the stroma were computed using an all-in-one fluorescence microscope BZ-X-700 with a hybrid cell count application BZ-H3C (Keyence, Osaka, Japan) [20] (Fig. 2D, E). Imaging analysis of the area ratio was employed because manual counting of TAMs was difficult.

### Setting cut-off values

Using all 107 cases, receiver operating characteristic (ROC) curves were drawn to determine the optimal cut-off values of quantitative TILs and TAMs by correlating their levels with recurrence or progression. Quantitative TILs and TAMs statuses were dichotomized according to the ROC thresholds.

### Statistical analysis

Recurrence-free survival (RFS) was considered as the period from the day of curative surgery until the day of documented recurrence in stage IB and IIIC patients; progression-free survival (PFS) was the period from the day of non-curative surgery until the day of documented progression of disease in stage IVB patients. Univariate and multivariate analyses were performed by the Cox’s proportional hazard general linear model. Multivariate analyses incorporated univariably significant parameters. Only one representative TIL-related factor was included in the multivariate analysis. The semiquantitatively and quantitatively evaluated TIL factors were separately analyzed. RFS and PFS curves were drawn using the Kaplan-Meier method and compared by the log-rank test. The clinicopathological implications of TILs and TAMs were analyzed by the chi-squared test or Fisher exact test. Two-sided *P* values of < 0.05 were considered as statistically significant. All statistical analyses were performed with JMP Pro 14 for Windows (SAS Institute, Inc., Cary, NC, USA).

## Results

The clinicopathological characteristics of the patients are summarized in Table 1. The median follow-up periods for patients other than those who died from EEC were 7.3 years (minimum to maximum 1.2–23.3 years) in the stage IB group and 6.5 years (minimum to maximum 0.25–19.3 years) in the stage IIIC/IVB group. In the IB group, 12 patients (20%) suffered from recurrence, and 5 (8.3%) died from EEC. In IIIC/IVB group, recurrence or progression occurred in 28 patients (60%), and 15 patients (32%) died from EEC.

**Table 1:** Clinicopathological features of endometrioid-type endometrial carcinoma patients (*n* = 107)

Parameter	Number of cases (%)					
	Total		Stage IB		Stage IIIC/IVB	
	*n* = 107 (100)		*n* = 60 (100)		*n* = 47 (100)	
Age (years)						
mean ± SD [minimum to maximum]	62.7 ± 11.0	[33–87]	65.2 ± 10.7	[33–87]	59.4 ± 10.7	[38–84]
≤ 50	11	(10)	3	(5)	8	(17)
> 50	96	(90)	57	(95)	39	(83)
Histological grade						
G1	40	(37)	28	(47)	12	(26)
G2	38	(36)	22	(37)	16	(34)
G3	29	(27)	10	(17)	19	(40)
Lymphovascular invasion						
Positive	80	(75)	39	(65)	41	(87)
Negative	27	(25)	21	(35)	6	(13)
Lymph node metastasis						
Positive	42	(39)	0	(0)	42	(89)
Negative	65	(61)	60	(100)	5	(11)
Surgical therapy						
Total hysterectomy + BSO	104	(97)	59	(98)	45	(96)
Total hysterectomy + LSO	1	(1)	1	(2)	0	(0)
Supracervical hysterectomy+ BSO	1	(1)	0	(0)	1	(2)
Supracervical hysterectomy + LSO	1	(1)	0	(0)	1	(2)
Adjuvant therapy						
Chemotherapy	79	(74)	43	(72)	36	(77)
Radiation	4	(4)	2	(3)	2	(4)
Radiation followed by chemotherapy	7	(6)	1	(2)	6	(13)
Not done	17	(16)	14	(22)	3	(6)

*BSO* Bilateral salpingo-oophorectomy, *LSO* Left salpingo-oophorectomy, *SD* Standard deviation

### Cut-off values for quantitative TIL and TAM evaluation

From the ROC curves for the 107 patients, the optimal cut-off values for CD3^+^ E-TILs, CD8^+^ E-TILs, CD3^+^ S-TILs, CD8^+^ S-TILs, CD68^+^ TAMs, and CD163^+^ TAMs were 3.0/HPF, 3.7/HPF, 59.8/HPF, 56.4/HPF, 0.78%, and 3.56%, respectively (Supplementary Table 1).

### Clinicopathological significance of TILs in the 60 stage IB EECs

According to semiquantitative evaluations, the densities of CD3^+^ E-TILs, CD8^+^ E-TILs, CD3^+^ S-TILs, and CD8^+^ S-TILs were high or intermediate in 37 (62%), 34 (57%), 36 (60%), and 33 (55%) of cases, respectively, in stage IB EECs (Table 2). Correlations were observed between CD8^+^ E-TILs and MMR deficiency (*P* = 0.0097), between CD3^+^ S-TILs and LVI (*P* = 0.011), between CD8^+^ S-TILs and grade (*P* = 0.014) and between CD8^+^ S-TILs and LVI (*P* = 0.013). The “TIL score” was not correlated with any clinicopathological parameters.

**Table 2:**
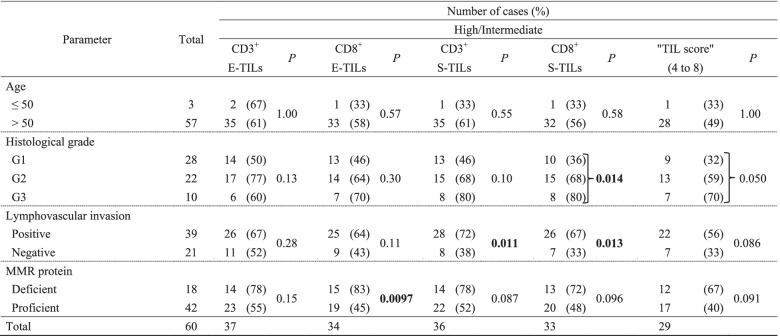
Correlations of semiquantitative high/intermediate TILs with clinicopathological parameters in stage IB endometrioid-type endometrial carcinoma (*n* = 60)

*P* values were computed by Chi-squared test, or Fisher’s exact test

*E-TILs* Epithelial tumor-infiltrating lymphocytes, *MMR* Mismatch repair, *S-TILs* Stromal tumor-infiltrating lymphocytes

According to quantitative evaluations, CD3^+^ E-TILs, CD8^+^ E-TILs, CD3^+^ S-TILs, and CD8^+^ S-TILs were high in 36 (60%), 26 (43%), 37 (62%), and 18 (30%) of the 60 stage IB EECs, respectively (Table 3). Correlations were detected between CD3^+^ E-TILs and grade (*P* = 0.035), CD8^+^ E-TILs and grade (*P* = 0.0040), between CD8^+^ S-TILs and grade (*P* = 0.049), and between CD8^+^ S-TILs and LVI (*P* = 0.017).

**Table 3:**
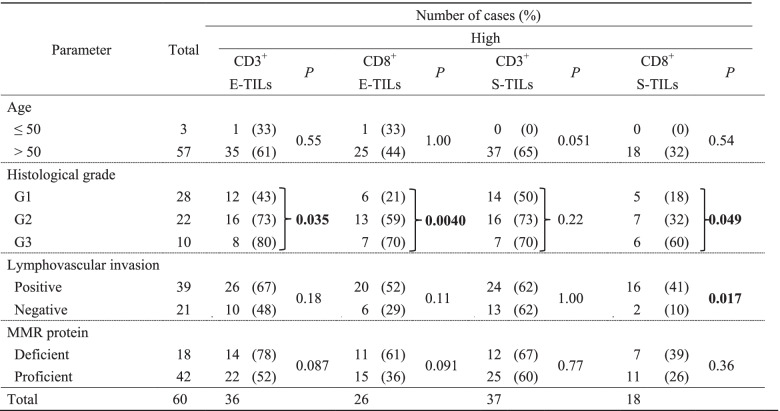
Correlations of quantitatively high TILs with clinicopathological parameters in stage IB endometrioid-type endometrial carcinoma (*n* = 60)

*P* values were calculated by chi-squared test or Fisher exact test

*E-TILs* Epithelial tumor-infiltrating lymphocytes, *MMR* Mismatch repair; *S-TILs* Stromal tumor-infiltrating lymphocytes

### Clinicopathological significance of TILs in the 47 stage IIIC/IVB EECs

Through semiquantitative evaluations, high/intermediate CD3^+^ E-TILs, CD8^+^ E-TILs, CD3^+^ S-TILs, and CD8^+^ S-TILs in stage IIIC/IVB EECs were detected in 24 (51%), 23 (49%), 26 (55%), and 20 (43%) of cases, respectively (Table 4). All TIL parameters were not correlated with any clinicopathological parameters.

**Table 4:** Correlations of semiquantitative high/intermediate TILs/“TIL score” with clinicopathological parameters in stage IIIC/IVB endometrioid-type endometrial carcinoma (*n* = 47)

Parameter	Total	Number of cases (%)														
		High/Intermediate														
		CD3^+^		*P*	CD8^+^		*P*	CD3^+^		*P*	CD8^+^		*P*	“TIL score”		*P*
		E-TILs			E-TILs			S-TILs			S-TILs			(4 to 8)		
Age																
≤ 50	8	3	(28)	0.46	3	(28)	0.70	3	(28)	0.43	3	(28)	1.00	2	(25)	0.69
> 50	39	21	(54)		20	(51)		23	(59)		17	(44)		14	(36)	
Stage																
IIIC	37	20	(53)	0.49	21	(57)	0.072	21	(57)	0.73	18	(49)	0.15	14	(38)	0.45
IVB	10	4	(40)		2	(20)		5	(50)		2	(20)		2	(20)	
Histological grade																
G1	12	5	(42)	0.67	5	(42)	0.83	7	(58)	0.94	4	(33)	0.38	2	(17)	0.28
G2	16	8	(50)		8	(50)		9	(56)		9	(56)		7	(44)	
G3	19	11	(53)		10	(53)		10	(53)		7	(37)		7	(37)	
Lymphovascular invasion																
Positive	41	20	(49)	0.66	19	(46)	0.41	21	(51)	0.20	16	(39)	0.37	13	(32)	0.39
Negative	6	4	(67)		4	(67)		5	(83)		4	(67)		3	(50)	
Lymph node metastasis																
Positive	42	22	(52)	0.66	21	(50)	1.00	24	(57)	0.64	19	(45)	0.37	14	(33)	1.00
Negative	5	2	(40)		2	(40)		2	(40)		1	(20)		2	(40)	
MMR protein																
Deficient	18	9	(50)	1.00	8	(44)	0.76	10	(56)	1.00	8	(44)	1.00	5	(28)	0.54
Proficient	29	15	(52)		15	(52)		16	(55)		12	(41)		11	(38)	
Total	47	24			23			26			20			16		

*P* values were calculated by chi-squared test or Fisher exact test

*E-TILs* Epithelial tumor-infiltrating lymphocytes, *MMR* Mismatch repair, *S-TILs* Stromal tumor-infiltrating lymphocytes

Through quantitative evaluations, CD3^+^ E-TILs, CD8^+^-E-TILs, CD3^+^ S-TILs, and CD8^+^ S-TILs were found to be high in 22 (47%), 15 (32%), 28 (60%), and 12 (26%) of cases, respectively (Table 5). High CD3^+^ E-TILs and CD8^+^ E-TILs analysed by quantitative evaluation were inversely correlated with LVI (*P* = 0.0069 and 0.0094, respectively).

**Table 5:** Correlations of quantitatively high TILs with clinicopathological parameters in stage IIIC/IVB endometrioid-type endometrial carcinoma (*n* = 47)

Parameter	Total	Number of patients (%)											
		Quantitatively high TILs											
		CD3^+^		*P*	CD8^+^		*P*	CD3^+^		*P*	CD8^+^		*P*
		E-TILs			E-TILs			S-TILs			S-TILs		
Age													
≤ 50	8	2	(25)	0.25	1	(13)	0.40	3	(38)	0.23	2	(25)	1.00
> 50	39	20	(51)		14	(36)		25	(64)		10	(26)	
Stage													
IIIC	37	18	(49)	0.72	11	(30)	0.70	23	(62)	0.49	10	(27)	1.00
IVB	10	4	(40)		4	(40)		5	(50)		2	(20)	
Histological grade													
G1	12	7	(58)	0.54	6	(50)	0.075	8	(67)	0.83	3	(25)	0.99
G2	16	6	(38)		2	(13)		9	(56)		4	(25)	
G3	19	9	(47)		7	(37)		11	(58)		5	(26)	
Lymphovascular invasion													
Positive	41	16	(39)	**0.0069**	10	(24)	**0.0094**	23	(56)	0.37	10	(24)	0.63
Negative	6	6	(100)		5	(83)		5	(83)		2	(33)	
Lymph node metastasis													
Positive	42	20	(48)	1.00	13	(31)	1.00	25	(60)	1.00	11	(26)	1.00
Negative	5	2	(40)		2	(40)		3	(69)		1	(20)	
MMR protein													
Deficient	18	9	(50)	0.77	7	(39)	0.52	13	(72)	0.22	5	(28)	1.00
Proficient	29	13	(45)		8	(28)		15	(52)		7	(24)	
Total	47	22			15			28			12		

*P* values were calculated by chi-squared test or Fisher exact test

*E-TILs* Epithelial tumor-infiltrating lymphocytes, *MMR* Mismatch repair, *S-TILs* Stromal tumor-infiltrating lymphocytes

MMR protein statuses were not associated with TILs or the “TIL score”.

### Prognostic significance of TILs in the 60 stage IB EECs

**Fig. 3:**
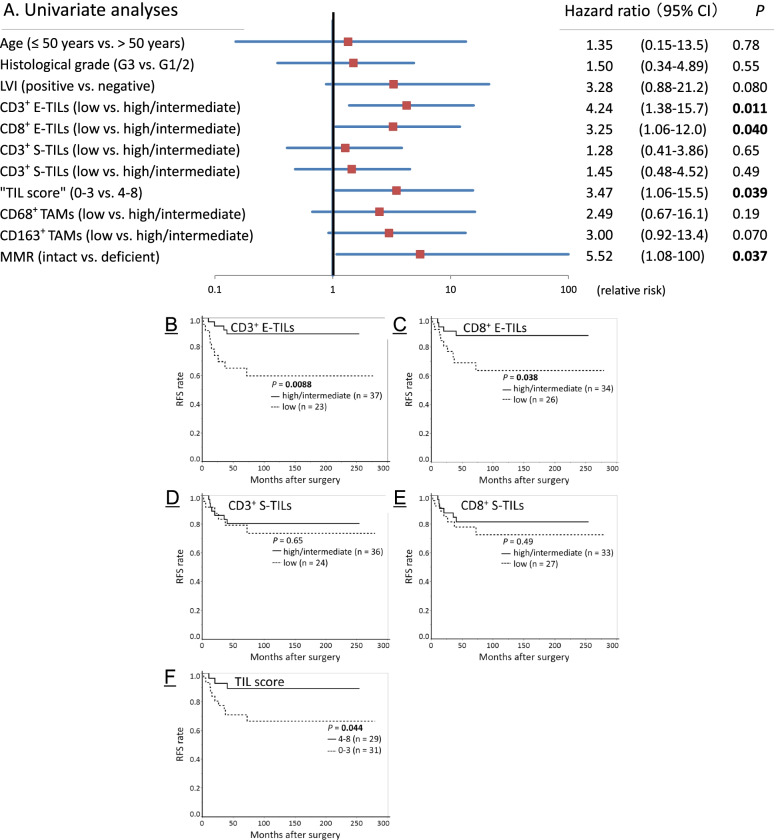
Recurrence-free survival (RFS) analyses of 60 stage IB endometrioid-type endometrial carcinoma. **A** Forest plot of Cox univariate analyses. Horizontal line: risk in logarithm. **B**-**F** RFS curves. Curves stratified by semiquantitative **B** CD3^+^ E-TILs, **C** CD8^+^ E-TILs, **D** CD3^+^ stromal TILs (S-TILs), **E** CD8^+^ S-TILs, and **F** “TIL score”. There were significant differences in the RFS curves between the high/intermediate and low TILs groups for CD3^+^ E-TILs and CD8^+^ E-TILs (*P* = 0.0088 and 0.038, respectively), and between high (score 4–8) and low (0–3) “TIL score” groups (*P* = 0.044). LVI, lymphovascular invasion; MMR, mismatch repair

In Cox univariate analyses, MMR proficiency (HR: 5.52, 95% CI: 1.08–100, *P* = 0.037), semiquantitative low CD3^+^ E-TILs (HR: 4.24, 95% CI: 1.38–15.7, *P* = 0.011), semiquantitative low CD8^+^ E-TILs (HR: 3.25, 95% CI: 1.06–12.0, *P* = 0.040), and low “TIL score” (HR: 3.47, 95% CI: 1.06–15.5, *P* = 0.039) were significant risk factors for recurrence (Fig. 3A).

In multivariate analysis including semiquantitative CD3^+^ E-TILs and MMR, only CD3^+^ E-TILs had an independent impact on RFS (*P* = 0.030) (Data not shown). When semiquantitative CD8^+^ E-TILs or the “TIL score” was included in multivariate analysis, instead of CD3^+^ E-TILs, the independent impact on RFS was not observed (data not shown). RFS curves significantly differed between high/intermediate and low TILs groups for semiquantitative CD3^+^ E-TILs (*P* = 0.0088) and CD8^+^ E-TILs (*P* = 0.038) and between the 2-tiered “TIL score” (score 0–3 vs. 4–8) groups (*P* = 0.044) (Fig. 3C–G).

Quantitative evaluations of the TILs showed no significant differences in the RFS curves although quantitative CD3^+^ and CD8^+^ E-TILs were nearly correlated with RFS (*P* = 0.071 and 0.10, respectively) (Supplementary Fig. 1).

### Prognostic significance of TILs in the 47 stage IIIC/IVB EECs

**Fig. 4:**
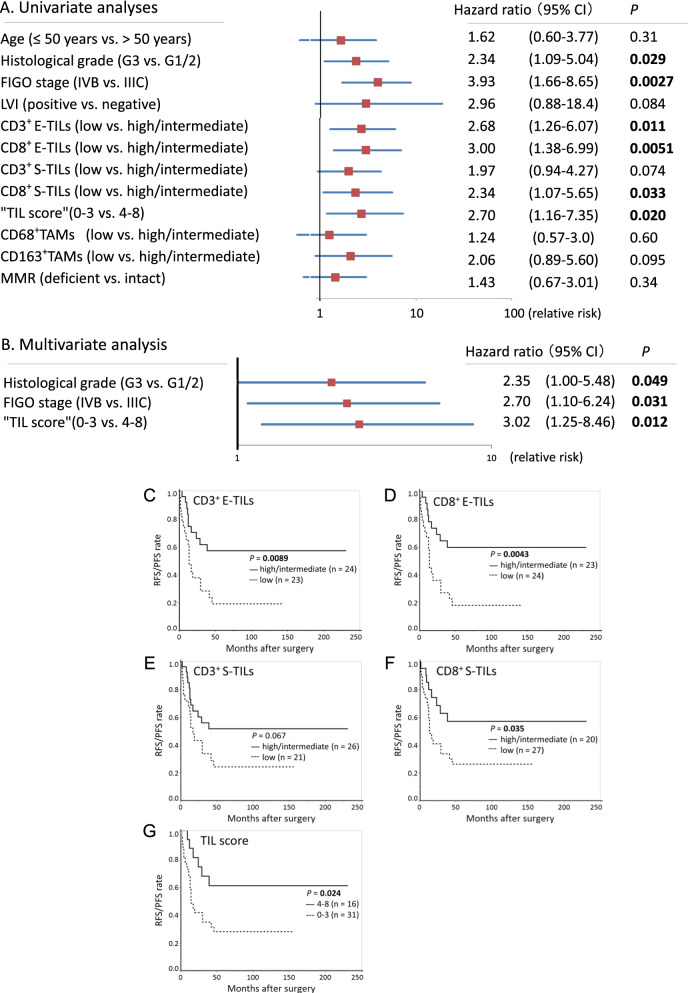
Survival analyses using semiquantitative TILs and TAMs in 47 stage IIIC/IVB endometrioid-type endometrial carcinoma. **A** Forest plot of Cox univariate analyses. **B** Forest plot of multivariate analysis. Multivariate analysis incorporates univariably significant parameters and “TIL score” as representative of semiquantitative TILs indicators. Vertical line: risk in logarithm. **C**–**G** Recurrence-free survival/progression-free survival curves. Curves stratified by semiquantitative (**C**) CD3^+^ E-TILs, (**D**) CD8^+^ E-TILs, (**E**) CD3^+^ stromal TILs (S-TILs), (**F**) CD8^+^ S-TILs, and (**G**) “TIL score”. There were significant differences in the curves between the high/intermediate and low CD3^+^ E-TILs, CD8^+^ E-TILs and CD8^+^ S-TILs groups (*P* = 0.0089, 0.0043 and 0.035, respectively) and between high (score 4–8) and low (0–3) “TIL score” groups (*P* = 0.024). LVI, lymphovascular invasion; MMR, mismatch repair

According to Cox univariate analyses, FIGO stage [HR: 3.93, 95%CI: 1.66–8.65, *P* = 0.0027] and grade 3 (HR: 2.34, 95%CI: 1.09–5.04, *P* = 0.029) were significant prognostic factors for RFS/PFS. In addition, semiquantitatively low CD3^+^ E-TILs (HR: 2.68, 95% CI: 1.26–6.07, *P* = 0.011), low CD8^+^ E-TILs (HR:3.00, 95% CI: 1.38–6.99, *P* = 0.0051), and low CD8^+^ S-TILs (HR: 2.34, 95%CI: 1.07–5.65, *P* = 0.033), and low “TIL score” (HR: 2.70, 95% CI: 1.16–7.35, *P* = 0.020) were also significant indicators of worse prognosis (Fig. 4A).

In multivariate analysis including stage, “TIL score” and grade, all these three had independent impact on RFS/PFS (*P* = 0.031, 0.012, and 0.049, respectively) (Fig. 4B). When semiquantitative CD3^+^ E-TILs and CD8^+^ E-TILs were included in the analyses, instead of the “TIL score”, independent impacts on RFS/PFS were also observed (*P* = 0.0011 and 0.0053, respectively); however, when semiquantitative CD8^+^ S-TILs was included in the analysis, independent impact was not observed (data not shown).

Based on the semiquantitative evaluation methods, there were significant differences in the RFS/PFS curves between the high/intermediate and low groups for CD3^+^ E-TILs, CD8^+^ E-TILs, and CD8^+^ S-TILs (*P* = 0.0089, 0.0043 and 0.035, respectively) (Fig. 4C, D, F). There was also significant differences in the RFS/PFS curves between the high and low “TIL score” (score 0–3 vs 4–8) groups (*P* = 0.024) (Fig. 4G).

**Fig. 5:**
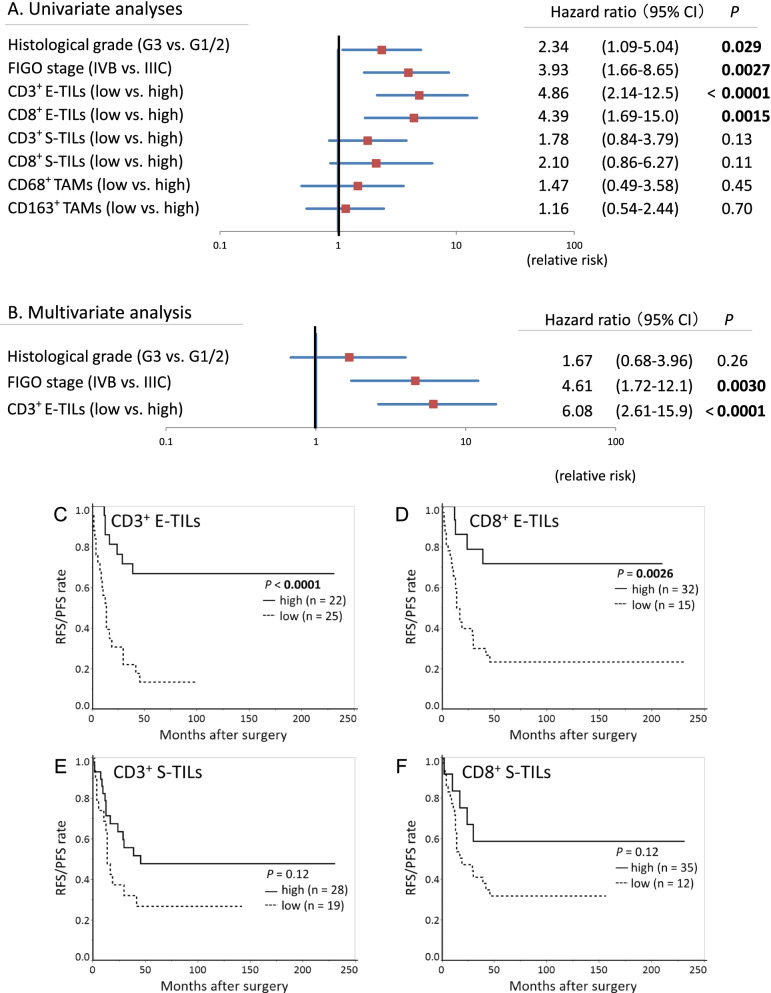
Survival analyses using quantitative TILs and TAMs in 47 stage IIIC/IVB endometrioid-type endometrial carcinoma. **A** Forest plot of Cox univariate analyses. **B** Forest plot of Cox multivariate analysis. Multivariate analysis incorporates univariably significant parameters and CD3^+^ E-TILs as representative of quantitative TILs indicators. Horizontal line: risk in logarithm. **C**–**F** Recurrence-free survival/progression-free survival curves. Curves stratified by quantitative (**C**) CD3^+^ E-TILs, (**D**) CD8^+^ E-TILs, (**E**) CD3^+^ stromal TILs (S-TILs), and (**F**) CD8^+^ S-TILs. There were significant differences in the curves between the high and low TILs groups for CD3^+^ E-TILs and CD8^+^ E-TILs (*P* < 0.0001 and 0.0026, respectively)

Univariate analyses revealed that quantitatively low CD3^+^ E-TILs (HR: 4.86, 95%CI: 2.14–12.5, *P* < 0.0001) and quantitatively low CD8^+^ E-TILs (HR: 4.39, 95%CI: 1.69–15.0, *P* = 0.0015) were also indicators of significantly worse prognosis (Fig. 5A). In Cox multivariate analysis including FIGO stage, quantitatively low CD3^+^ E-TILs, and grade 3, the former two were independent risk factors for recurrence/progression (*P* = 0.0030 and < 0.0001, respectively) (Fig. 5B). When quantitative CD8^+^ E-TILs were included in the analysis, instead of quantitative CD3^+^ E-TILs, independent impact on RFS/PFS was also observed (*P* < 0.0001) (Data not shown).

Based on quantitative evaluation, there were significant differences in the RFS/PFS curves between the high and low groups for both CD3^+^ E-TILs and CD8^+^ E-TILs (*P* < 0.0001 and 0.0026, respectively), whereas differences were not observed for CD3^+^ S-TILs or CD8^+^ S-TILs (*P =* 0.12 and 0.12, respectively) (Fig. 5C–F).

### Clinicopathological and prognostic significance of TAMs

Semiquantitative or quantitative CD68^+^ or CD163^+^ T AMs were not correlated with any clinicopathological parameters or MMR statuses in the stage IB or stage IIIC/IVB patient groups (Supplementary Fig. 2A-H). They also showed no correlation with RFS/PFS in both early and advanced stage patients (Supplementary Tables 2 and 3).

## Discussion

We examined the prognostic and clinicopathological implications of TILs and TAMs in patient groups with EEC in both early stages (FIGO IB) and advanced stages (FIGO IIIC and IVB). In 60 patients with stage IB disease, semiquantitatively low CD3^+^ E-TILs, and low CD8^+^ E-TILs, and low “TIL score” were correlated with lower RFS rates. Similarly, in 47 patients with stages IIIC/IVB disease, low CD3^+^ E-TILs and low CD8^+^ E-TILs, evaluated by semiquantitative and quantitative methods, and a low “TIL score” were correlated with a worse prognosis. Based on these results, low E-TILs may be useful biomarkers for worse clinical outcomes in patients in early stages, whereas high E-TILs were shown to indicate a better prognosis in patients in advanced stages. In contrast, S-TILs, CD68^+^ TAMs, and CD163^+^ TAMs evaluated by all methods were not correlated with the prognosis of patient groups at both early and advanced clinical stages, except for semiquantitative CD8^+^ S-TILs in the IIIC/IVB group.

In semiquantitative evaluation, the density of E-TILs was categorized as low, intermediate, and high. On their evaluation, we need to consider whether the number of immunopositive lymphocytes within the tumor cell nests was > 3 per HPF (× 400, approximately 0.238 mm^2^) or not. With this evaluation, both CD8^+^ E-TILs and CD3^+^ E-TILs were strongly correlated with the better prognosis of patients in both early and advanced stages.

Similarly, in semiquantitative evaluation, the density of S-TILs in the invasive front was classified as low, intermediate, or high. This classification is similar to that used by Yamashita et al. [19]. They classified the density of CD8^+^ TILs, including both S-TILs and E-TILs, as low (0–30%), moderate (30–60%), and high (> 60%), and moderate/high CD8^+^ TILs were correlated with a higher PFS rate in 141 patients with EC [19]. Using the present evaluation method, we observed a significant relationship of CD8^+^ S-TILs with higher RFS/PFS rates only in the stage IIIC/IVB EEC group, but their prognostic impact was weaker than those of CD8^+^/CD3^+^ E-TILs.

In quantitative evaluation, the cut-off values of high TILs varied among studies, ranging from 7.065 to 25 per × 200 field (0.785 mm^2^) for CD8^+^ E-TILs [8–11] and from 13.345 to 35 cells per × 200 field for CD3^+^ E-TILs [8, 9]. Hendry et al. evaluated TILs by using a cut-off value of 40 lymphocytes per 10 HPFs on haematoxylin and eosin-stained slides, approximately corresponding to 16 cells per × 200 field [21]. In the present study, the thresholds for CD8^+^ E-TILs and CD3^+^ E-TILs were set to 14.8 and 12.0 cells per × 200 field, respectively. These values were within or near the ranges of the thresholds. Based on these values, these E-TILs were significantly correlated with prognosis in the stage IIIC/IVB group and showed marginal prognostic significance in the stage IB group.

The depth of invasion and status of the invasive front of the primary site are important determinants of clinical outcomes of EC as well as colorectal cancer. The TIL status there would be a parameter of strength of in situ adaptive immune reaction at the primary site. The “Immunoscore” evaluated a combination of CD3^+^ and CD8^+^ TILs in both the tumor center and invasive front and showed the greatest impact on the risk of recurrence and death among the clinical parameters of colorectal cancer [11, 12]. Because our digital pathology environment was incomplete, we employed a surrogate “TIL score” by summing the semiquantitative points of CD3^+^ E-TILs, CD8^+^ E-TILs, CD3^+^ S-TILs, and CD8^+^ S-TILs. This “TIL score” was a significant predictor of prognosis in both patients with stage IB and stage IIIC/IVB disease, but the combination did not appear to dramatically increase the prognostic impact compared with simple measurement of CD3^+^ E-TILs or CD8^+^ E-TILs.

TAMs play important roles in immunity and the tumor microenvironment. CD68 and CD163 have been used as immunohistochemical markers of these cells. Soeda et al. classified TAMs that infiltrated into the cancer nests or stroma along the tumor-myometrial junction (margin TAM) into high and low levels based on a threshold of 20 cells per × 200 field (0.785 mm^2^) and observed the prognostic significance of high-level CD68^+^ TAMs [15]. Kübler et al. and Espinosa et al. also showed that CD163^+^ TAMs were correlated with worse prognosis and/or regional lymph node metastases in patients with EECs [22, 23]. Espinosa et al. scored the density of CD163-positive cells as 0, 1, and 2 when the number of cells was ≤20, > 20 to ≤50, and > 50 per 1-mm diameter core (0.785 mm^2^) [23], whereas Kübler et al. set the median value of immunostaining as a cut-off point between high and low TAM counts [22].

We examined both CD68^+^ and CD163^+^ cells using two different methods but did not find significant correlations of TAM density with clinicopathological parameters or with patient prognosis in early or advanced stage EECs. Although there were no similar results, the present findings may be explained as follows: As the cancer progresses, cancer-associated fibroblasts begin to continuously release growth factors such as TGF-β (transforming growth factor-β) and SDF-1/CXCL12 (stromal derived factor-1/C-X-C motif chemokine ligand 12), which chemically attract macrophages and promote M2 polarization [24] and, in parallel, modulate the extracellular matrix. TAMs are preferably attracted to hyaluronan, which is a major component of the extracellular matrix. Depletion of hyaluronan synthase 2 in cancer-associated fibroblasts reduces TAM recruitment and thereby attenuates tumor angiogenesis and lymphangiogenesis [24]. Therefore, even in EECs at advanced stages, the number of TAMs may not increase compared to in EECs at earlier stages. To clarify the reproducibility of the present results for TAMs in EEC, further studies of a much larger cohort are necessary.

Interestingly, the clinicopathological implications of TILs somewhat differed between the early and advanced stages of EEC. S-TILs tended to be correlated with LVI in the IB group but E-TILs were inversely correlated with LVI in the IIIC/IVB group. The relationship between TILs and MMR deficiency also appeared to differ between early and advanced stage EECs. In the present study, only the stage IB EEC group showed a tendency for a relationship between MMR deficiency and higher CD8^+^ E-TILs and better prognosis. Deficient MMR is detected in approximately 30% of EC cases [25] and was shown to be correlated with CD8^+^ TILs in previous studies [10, 26, 27]; however, its prognostic significance is controversial [10, 26–30]. The relationship of the TIL status with the grade, LVI, and MMR status in EECs appeared to significantly differ between early and advanced stages. Such differences may derive from the status of tumor microenvironment of tumor stroma.

TILs were established to be effective markers of better prognosis and response to chemotherapy in breast cancer, melanoma, and colorectal cancers. Recently, the Immunoscore was also shown to be a useful marker for better prognosis and efficacy of primary chemotherapy in esophageal cancer [31]. In ovarian cancer, as another gynecological cancer, higher number of CD3^+^ and CD8^+^ E-TILs, with the threshold of > 10 TILs/HPF, was shown to be associated with a good prognosis although there are no standardized TIL measurement method [32].

Limitations of the present study include the manual evaluation methods and relatively small number of cases. First, quantitative evaluations of TILs were performed using digital images from several microscopic HPFs. Particularly, because of a relatively heterogeneous distribution of S-TILs, selection bias of the evaluation fields may have affected the results and caused underestimation of the prognostic impact of S-TILs. The conditions appeared similar in the evaluation of TAMs. Second, the numbers of stage IB, IIIC, and IVB cases were small. Specifically, the number of events in stage IB cases may not have adequate detection power to reveal the prognostic implication of TILs or TAMs. A large-scale study using a simpler, more accurate, and well standardized imaging analysis is required to establish the prognostic roles of these cells in early EECs.

## Conclusion

Semiquantitative E-TILs were suggested to be representative as the TILs that are correlated with prognosis in both early and advanced stage patients with EEC. Particularly, CD3^+^ E-TILs and CD8^+^ E-TILs were shown to be useful markers of better prognosis in patients with EEC, regardless of the stage.

## Supplementary Information

**Additional file 1. Supplementary Table 1.** Quantitative TILs and TAMs and cut-off values (*n* = 107).

**Additional file 2. Supplementary Fig. 1.** Recurrence-free survival curves of 60 stage IB endometrioid-type endometrial carcinoma. Curves were stratified by quantitative (A) CD3^+^ epithelial tumor-infiltrating lymphocytes (E-TILs), (B) CD8^+^ E-TILs, (C) CD3^+^ stromal TILs (S-TILs) and (D) CD8^+^ S-TILs. Curves were not significantly different.

**Additional file 3. Supplementary Fig. 2.** Survival analyses using semiquantative and quantative TAMs in endometrioid-type endometrial carcinoma. Recurrence-free survival (RFS) curves of 60 patients with stage IB EEC stratified by semiquantitative (A) CD68^+^ tumor associated macrophages (TAMs), (B) semiquantitative CD163^+^ TAMs, (C) quantitative CD68^+^ TAMs, and (D) quantitative CD163^+^ TAMs. Curves were not significantly different. RFS/progression-free survival (PFS) curves of 47 patients with stage IIIC/IVB EEC stratified by (E) semiquantitative CD68^+^ TAMs, (F) semiquantitative CD163^+^ TAMs, (G) quantitative CD68^+^ TAMs and (H) quantitative CD163^+^ TAMs. Curves were not significantly different.

**Additional file 4. Supplementary Table 2.** Correlations of semiquantitative high/intermediate CD68^+^ TAMs and CD163^+^ TAMs with clinicopathological parameters in stage IB and stage IIIC/IVB endometrioid-type endometrial carcinoma.

**Additional file 5. Supplementary Table 3.** Correlations of quantitative high CD68^+^ TAMs and CD163^+^ TAMs with clinicopathological parameters in stage IB and stage IIIC/IVB endometrioid-type endometrial carcinoma.

## Data Availability

Datasets used and/or analyzed during this study are available from the corresponding author on reasonable request.

## Abbreviations

CI: Confidence interval
CTL: Cytotoxic T lymphocyte
EC: Endometrial cancer
EEC: Endometrioid carcinoma of the endometrium, endometrioid-type endometrial carcinoma
E-TILs: Epithelial tumor-infiltrating lymphocytes
FIGO: International Federation of Gynecology and Obstetrics
HPF: High-power field
HR: Hazard ratio
LVI: Lymphovascular invasion
MMR: Mismatch repair
PFS: Progression-free survival
RFS: Recurrence-free survival
ROC: Receiver operating characteristic
SD: Standard deviation
S-TILs: Stromal tumor-infiltrating lymphocytes
TAMs: Tumor-associated macrophages
TILs: Tumor-infiltrating lymphocytes
