# Supplementary material for: Intraepithelial lymphocytes are indicators of better prognosis in surgically resected endometrioid-type endometrial carcinomas at early and advanced stages
Source: BMC Cancer. 2022 Apr 2;22:361. doi: 10.1186/s12885-022-09363-0 (PMC8977032; doi:10.1186/s12885-022-09363-0)
Supplement: Supplementary file 1 — Additional file 1. Supplementary Table 1. Quantitative TILs and TAMs and cut-off values (n = 107). [file 12885_2022_9363_MOESM1_ESM.docx]

| Supplementary table 1. Quantitative TILs and TAMs and cut-off values (n =107) | | | | |
| --- | --- | --- | --- | --- |
|  | | | | |
| Parameter | Median | Mean (±SD) | Cut-off value from ROC curve* | Area under ROC curve |
| CD3^+^ E-TILs | 3.3/HPF | 6.4/HPF (± 7.0) | 3.0/HPF | 0.710 |
| CD8^+^ E-TILs | 2.7/HPF | 4.7/HPF (± 6.0) | 3.7/HPF | 0.692 |
| CD3^+^ S-TILs | 71.8/HPF | 84.7/HPF (± 53.9) | 59.8/HPF | 0.537 |
| CD8^+^ S-TILs | 34.4/HPF | 43.8/HPF (± 36.5) | 56.4/HPF | 0.560 |
| CD68^+^ TAMs | 1.50% | 2.25% (± 2.24) | 0.78% | 0.549 |
| CD163^+^ TAMs | 3.72% | 4.52% (± 3.68) | 3.56% | 0.529 |

E-TILs, Epithelial tumor-infiltrating lymphocytes; HPF, High power fields; ROC, receiver operating characteristic; S-TILs, Stromal tumor-infiltrating lymphocytes; TAMs, Tumor associated macrophages
