# Supplementary figures and images for: Intraepithelial lymphocytes are indicators of better prognosis in surgically resected endometrioid-type endometrial carcinomas at early and advanced stages
Source: BMC Cancer. 2022 Apr 2;22:361. doi: 10.1186/s12885-022-09363-0 (PMC8977032; doi:10.1186/s12885-022-09363-0)

Supplementary Fig. 1

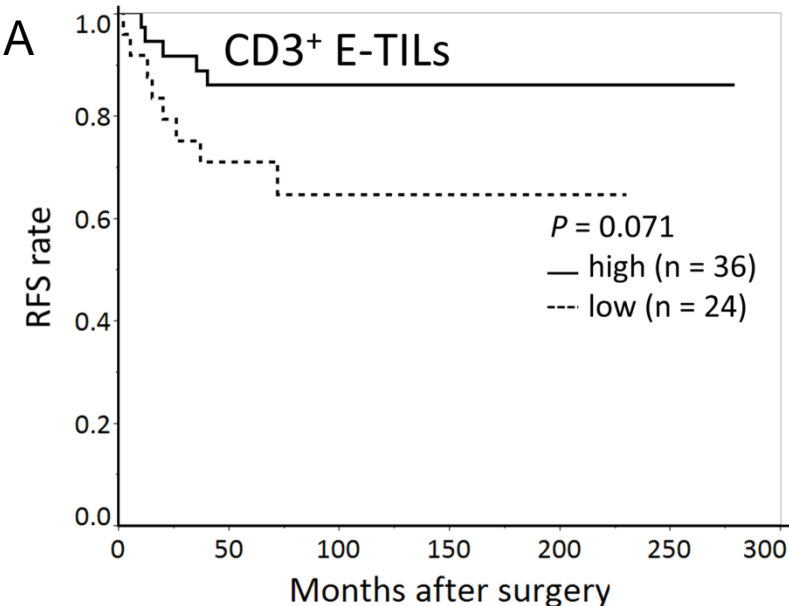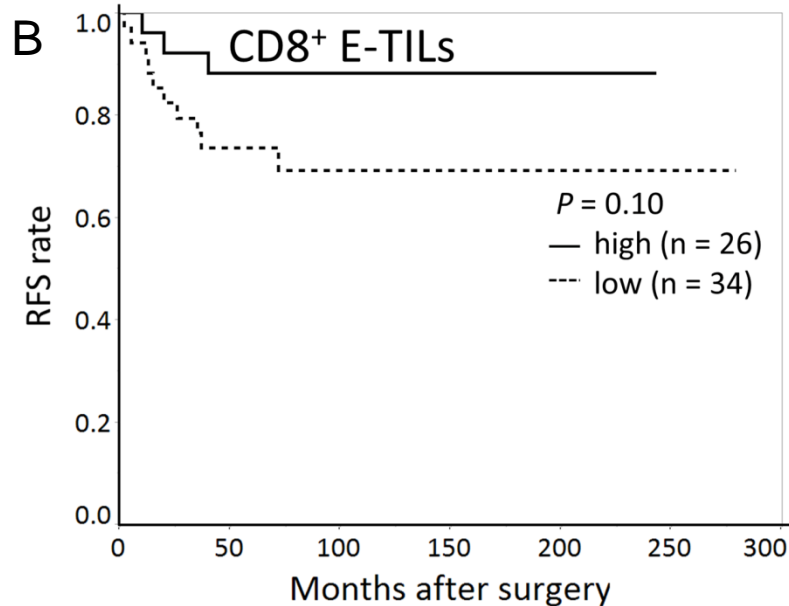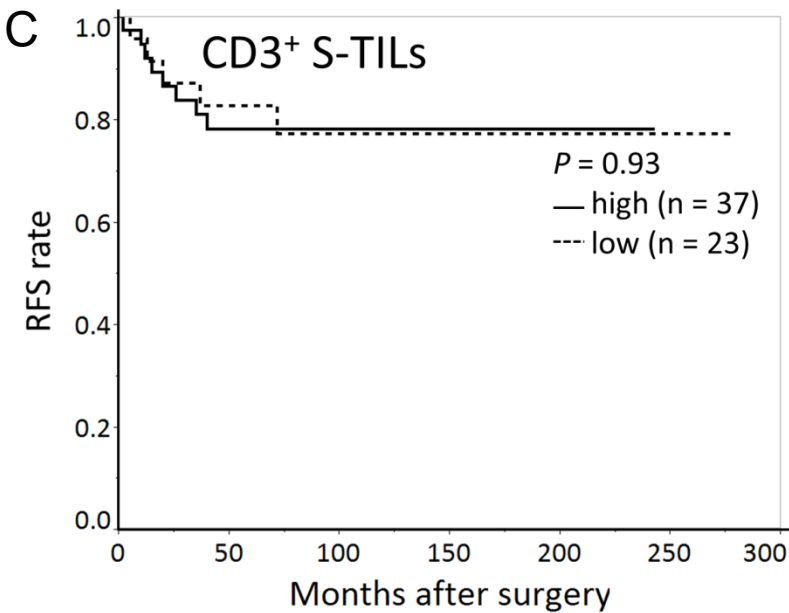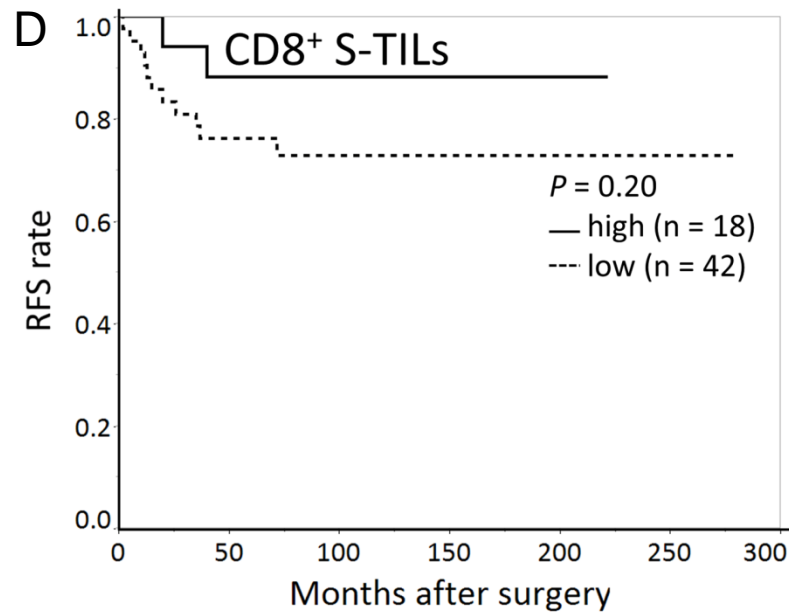

Supplement: Supplementary file 2 — Additional file 2. Supplementary Fig. 1. Recurrence-free survival curves of 60 stage IB endometrioid-type endometrial carcinoma. Curves were stratified by quantitative (A) CD3+ epithelial tumor-infiltrating lymphocytes (E-TILs), (B) CD8+ E-TILs, (C) CD3+ stromal TILs (S-TILs) and (D) CD8+ S-TILs. Curves were not significantly different. [file 12885_2022_9363_MOESM2_ESM.pdf]

Supplementary Fig. 2

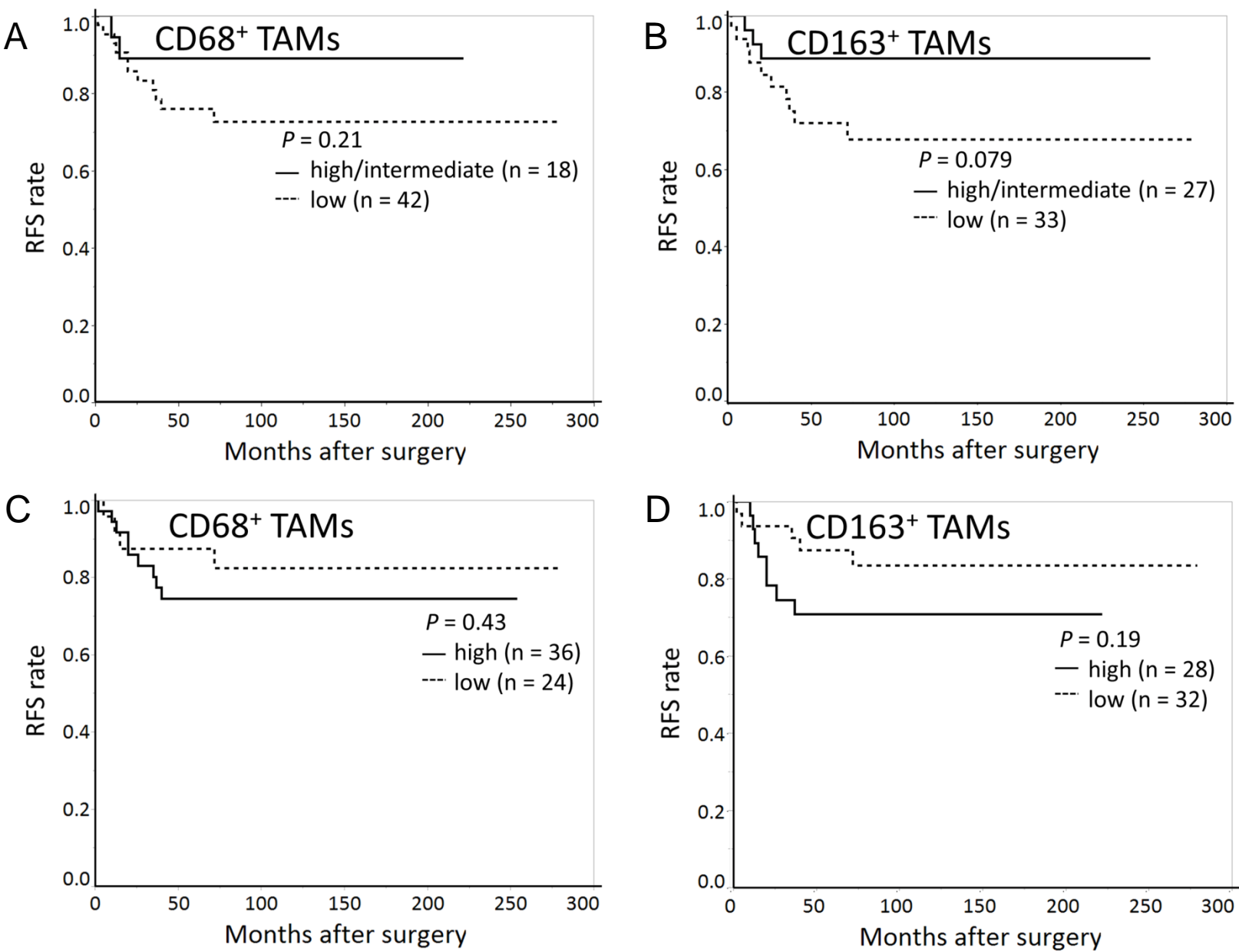

Supplementary Fig. 2

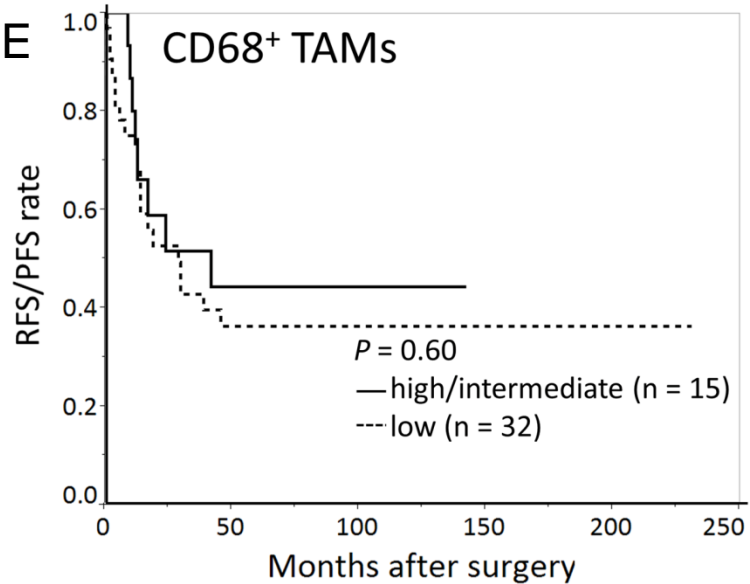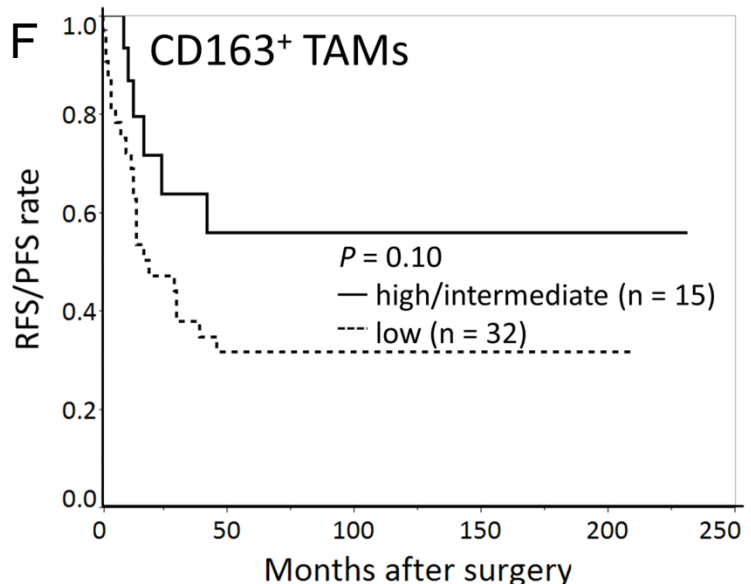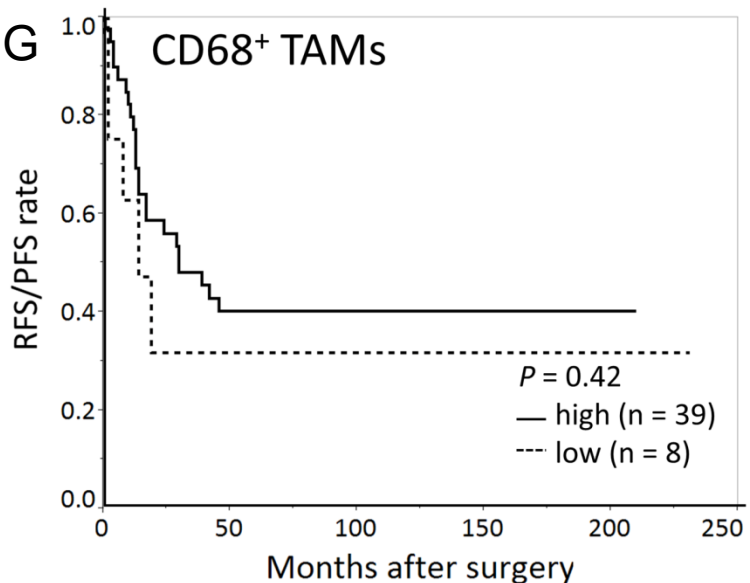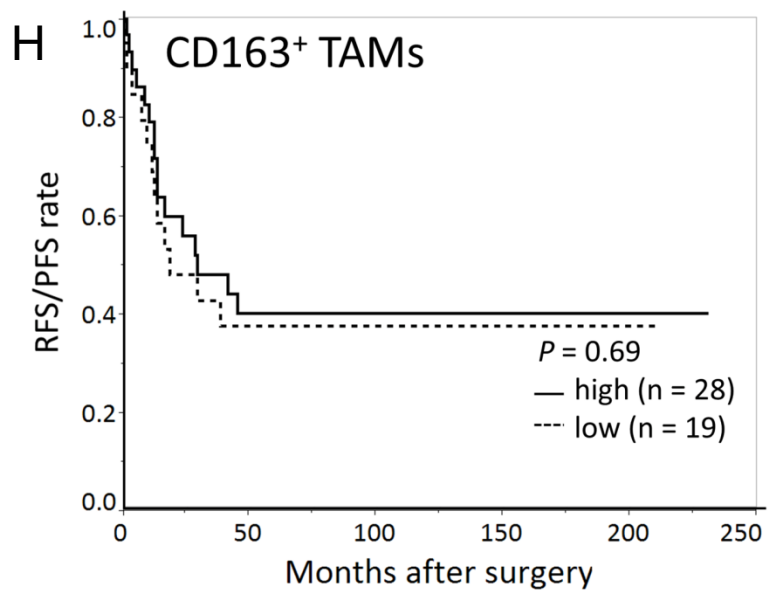

Supplement: Supplementary file 3 — Additional file 3. Supplementary Fig. 2. Survival analyses using semiquantative and quantative TAMs in endometrioid-type endometrial carcinoma. Recurrence-free survival (RFS) curves of 60 patients with stage IB EEC stratified by semiquantitative (A) CD68+ tumor associated macrophages (TAMs), (B) semiquantitative CD163+ TAMs, (C) quantitative CD68+ TAMs, and (D) quantitative CD163+ TAMs. Curves were not significantly different. RFS/progression-free survival (PFS) curves of 47 patients with stage IIIC/IVB EEC stratified by (E) semiquantitative CD68+ TAMs, (F) semiquantitative CD163+ TAMs, (G) quantitative CD68+ TAMs and (H) quantitative CD163+ TAMs. Curves were not significantly different. [file 12885_2022_9363_MOESM3_ESM.pdf]
