# Supplementary material for: Intraepithelial lymphocytes are indicators of better prognosis in surgically resected endometrioid-type endometrial carcinomas at early and advanced stages
Source: BMC Cancer. 2022 Apr 2;22:361. doi: 10.1186/s12885-022-09363-0 (PMC8977032; doi:10.1186/s12885-022-09363-0)
Supplement: Supplementary file 4 — Additional file 4. Supplementary Table 2. Correlations of semiquantitative high/intermediate CD68+ TAMs and CD163+ TAMs with clinicopathological parameters in stage IB and stage IIIC/IVB endometrioid-type endometrial carcinoma. [file 12885_2022_9363_MOESM4_ESM.docx]

| Supplementary table 2. Correlations of semiquantitative high/intermediate CD68^+^ TAMs and CD163^+^ TAMs with clinicopathological parameters in stage IB and stage IIIC/IVB endometrioid-type endometrial carcinoma | | | | | | | | | | | | | | | | | |
| --- | --- | --- | --- | --- | --- | --- | --- | --- | --- | --- | --- | --- | --- | --- | --- | --- | --- |
|  |  |  |  |  |  |  |  |  |  |  |  |  |  |  |  |  |  |
|  | Number of patients (%) | | | | | | | | | | | | | | | | |
| Parameter | Stage IB (n = 60) | | | | | | | |  | Stage IIIC/IVB (n = 47) | | | | | | | |
|  | Total | High/Intermediate | | | | | | |  | Total | High/Intermediate | | | | | | |
|  |  | CD68^+^ | | *P* |  | CD163^+^ | | *P* |  |  | CD68^+^ | | *P* |  | CD163^+^ | | *P* |
| Age |  |  |  |  |  |  |  |  |  |  |  |  |  |  |  |  |  |
| ≤ 50 | 3 | 1 | (33) | 1.00 |  | 1 | (33) | 1.00 |  | 8 | 5 | (63) | 0.089 |  | 4 | (50) | 0.24 |
| > 50 | 57 | 17 | (30) |  |  | 26 | (46) |  |  | 39 | 10 | (26) |  |  | 11 | (28) |  |
| Stage |  |  |  |  |  |  |  |  |  |  |  |  |  |  |  |  |  |
| IIIC |  |  |  |  |  |  |  |  |  | 37 | 11 | (30) | 0.70 |  | 12 | (32) | 1.00 |
| IVB |  |  |  |  |  |  |  |  |  | 10 | 4 | (40) |  |  | 3 | (30) |  |
| Histological grade |  |  |  |  |  |  |  |  |  |  |  |  |  |  |  |  |  |
| G1 | 28 | 9 | (32) | 0.18 |  | 12 | (43) | 0.92 |  | 12 | 3 | (25) | 0.77 |  | 2 | (17) | 0.14 |
| G2 | 22 | 4 | (18) |  |  | 10 | (45) |  |  | 16 | 6 | (38) |  |  | 8 | (50) |  |
| G3 | 10 | 5 | (50) |  |  | 5 | (50) |  |  | 19 | 6 | (32) |  |  | 5 | (26) |  |
| Lymphovascular invasion |  |  |  |  |  |  |  |  |  |  |  |  |  |  |  |  |  |
| Positive | 39 | 12 | (31) | 1.00 |  | 19 | (49) | 0.59 |  | 41 | 13 | (32) | 1.00 |  | 13 | (32) | 1.00 |
| Negative | 21 | 6 | (29) |  |  | 8 | (38) |  |  | 6 | 2 | (33) |  |  | 2 | (33) |  |
| Lymph node metastasis |  |  |  |  |  |  |  |  |  |  |  |  |  |  |  |  |  |
| Positive |  |  |  |  |  |  |  |  |  | 42 | 13 | (31) | 0.64 |  | 14 | (33) | 1.00 |
| Negative |  |  |  |  |  |  |  |  |  | 5 | 2 | (40) |  |  | 1 | (20) |  |
| MMR protein |  |  |  |  |  |  |  |  |  |  |  |  |  |  |  |  |  |
| Deficient | 18 | 7 | (39) | 0.36 |  | 11 | (61) | 0.16 |  | 18 | 6 | (33) | 1.00 |  | 5 | (28) | 0.75 |
| Proficient | 42 | 11 | (26) |  |  | 16 | (38) |  |  | 29 | 9 | (31) |  |  | 10 | (34) |  |
| Total | 60 | 18 |  |  |  | 27 |  |  |  | 47 | 15 |  |  |  | 15 |  |  |

*P* values were calculated by chi-squared test or Fisher exact test. MMR, Mismatch repair; TAMs, Tumor associated macrophages
