# Supplementary material for: Intraepithelial lymphocytes are indicators of better prognosis in surgically resected endometrioid-type endometrial carcinomas at early and advanced stages
Source: BMC Cancer. 2022 Apr 2;22:361. doi: 10.1186/s12885-022-09363-0 (PMC8977032; doi:10.1186/s12885-022-09363-0)
Supplement: Supplementary file 5 — Additional file 5. Supplementary Table 3. Correlations of quantitative high CD68+ TAMs and CD163+ TAMs with clinicopathological parameters in stage IB and stage IIIC/IVB endometrioid-type endometrial carcinoma. [file 12885_2022_9363_MOESM5_ESM.docx]

| Supplementary table 3. Correlations of quantitative high CD68^+^ TAMs and CD163^+^ TAMs with clinicopathological parameters in stage IB and stage IIIC/IVB endometrioid-type endometrial carcinoma | | | | | | | | | | | | | | | | | |
| --- | --- | --- | --- | --- | --- | --- | --- | --- | --- | --- | --- | --- | --- | --- | --- | --- | --- |
|  |  |  |  |  |  |  |  |  |  |  |  |  |  |  |  |  |  |
|  | Number of patients (%) | | | | | | | | | | | | | | | | |
| Parameter | Stage IB (n = 60) | | | | | | | |  | Stage IIIC/IVB (n = 47) | | | | | | | |
|  | Total | High | | | | | | |  | Total | High | | | | | | |
|  |  | CD68^+^ | | *P* |  | CD163^+^ | | *P* |  |  | CD68^+^ | | *P* |  | CD163^+^ | | *P* |
| Age |  |  |  |  |  |  |  |  |  |  |  |  |  |  |  |  |  |
| ≤ 50 | 3 | 2 | (67) | 1.00 |  | 1 | (33) | 1.00 |  | 8 | 6 | (75) | 0.60 |  | 5 | (86) | 1.00 |
| > 50 | 57 | 34 | (60) |  |  | 27 | (47) |  |  | 39 | 33 | (85) |  |  | 23 | (59) |  |
| Stage |  |  |  |  |  |  |  |  |  |  |  |  |  |  |  |  |  |
| IIIC |  |  |  |  |  |  |  |  |  | 37 | 31 | (84) | 1.00 |  | 24 | (65) | 0.28 |
| IVB |  |  |  |  |  |  |  |  |  | 10 | 8 | (80) |  |  | 4 | (40) |  |
| Histological grade |  |  |  |  |  |  |  |  |  |  |  |  |  |  |  |  |  |
| G1 | 28 | 14 | (50) | 0.058 |  | 9 | (32) | 0.073 |  | 12 | 10 | (83) | 0.52 |  | 4 | (33) | 0.076 |
| G2 | 22 | 13 | (59) |  |  | 12 | (55) |  |  | 16 | 12 | (75) |  |  | 12 | (75) |  |
| G3 | 10 | 9 | (90) |  |  | 7 | (70) |  |  | 19 | 17 | (89) |  |  | 12 | (63) |  |
| Lymphovascular invasion |  |  |  |  |  |  |  |  |  |  |  |  |  |  |  |  |  |
| Positive | 39 | 23 | (59) | 1.00 |  | 20 | (51) | 0.42 |  | 41 | 34 | (83) | 1.00 |  | 26 | (63) | 0.20 |
| Negative | 21 | 13 | (62) |  |  | 8 | (38) |  |  | 6 | 5 | (83) |  |  | 2 | (33) |  |
| Lymph node metastasis |  |  |  |  |  |  |  |  |  |  |  |  |  |  |  |  |  |
| Positive |  |  |  |  |  |  |  |  |  | 42 | 34 | (81) | 0.57 |  | 25 | (60) | 1.00 |
| Negative |  |  |  |  |  |  |  |  |  | 5 | 5 | (100) |  |  | 3 | (60) |  |
| MMR protein |  |  |  |  |  |  |  |  |  |  |  |  |  |  |  |  |  |
| Deficient | 18 | 11 | (61) | 1.00 |  | 10 | (56) | 0.40 |  | 18 | 14 | (78) | 0.69 |  | 10 | (56) | 0.76 |
| Proficient | 42 | 25 | (60) |  |  | 18 | (43) |  |  | 29 | 25 | (86) |  |  | 18 | (62) |  |
| Total | 60 | 18 |  |  |  | 27 |  |  |  | 47 | 39 |  |  |  | 28 |  |  |

*P* values were calculated by chi-squared test or Fisher exact test. MMR, Mismatch repair; TAMs, Tumor associated macrophages
